# Polygenic risk scores to stratify cancer screening should predict mortality not incidence

**DOI:** 10.1038/s41698-022-00280-w

**Published:** 2022-05-30

**Authors:** Andrew J. Vickers, Amit Sud, Jonine Bernstein, Richard Houlston

**Affiliations:** 1 Department of Epidemiology and Biostatistics, Memorial Sloan Kettering Cancer Center, New York, NY USA; 2 Division of Genetics and Epidemiology, The Institute of Cancer Research, London, UK

**Keywords:** Risk factors, Prostate cancer

## Abstract

Population-based cancer screening programs such as mammography or colonscopy generally directed at all healthy individuals in a given age stratum. It has recently been proposed that cancer screening could be restricted to a high-risk subgroup based on polygenic risk scores (PRSs) using panels of single-nucleotide polymorphisms (SNPs). These PRSs were, however, generated to predict cancer incidence rather than cancer mortality and will not necessarily address overdiagnosis, a major problem associated with cancer screening programs. We develop a simple net-benefit framework for evaluating screening approaches that incorporates overdiagnosis. We use this methodology to demonstrate that if a PRS does not differentially discriminate between incident and lethal cancer, restricting screening to a subgroup with high scores will only improve screening outcomes in a small number of scenarios. In contrast, restricting screening to a subgroup defined as high-risk based on a marker that is more strongly predictive of mortality than incidence will often afford greater net benefit than screening all eligible individuals. If PRS-based cancer screening is to be effective, research needs to focus on identifying PRSs associated with cancer mortality, an unchartered and clinically-relevant area of research, with a much higher potential to improve screening outcomes.

## Introduction

Population-based cancer screening programs are generally directed at all healthy individuals in a given age stratum. For instance, mammography screening is typically offered to women aged 45–70 and fecal blood tests or colonoscopy to all individuals at similar ages. In many countries, men aged 45–55 years are recommended to engage in a shared-decision making process with their doctor with respect to prostate-specific antigen (PSA) testing. It seems intuitive that the net benefit of these and other cancer screening programs will be improved by risk stratification, focusing screening on those at higher risk and screening less often, or minimally, those at lower risk.

Genome-wide association studies have identified associations between single nucleotide polymorphisms (SNPs) and the risk of developing many types of cancer^1^. Proponents assert that polygenic risk score (PRS) testing, based on panels of risk SNPs, will improve early detection of cancer through individualized screening programs. Enthusiasm for PRSs is well-documented in recent publications such as the Genome UK report^2^. Moreover, an increasing number of companies offer direct to consumers genetic testing that incorporate PRSs. Clinical studies, such as BARCODE1^3^ have been launched, whereby men with a prostate cancer PRS in the top decile of risk undergo prostate imaging and biopsy.

While PRS testing to risk-stratify screening is highly seductive, there have been concerns. These have largely focused on the questionable discriminatory capability of PRSs, low utility in less common cancers, and lack of replication in non-European populations^4,5^. Here we raise an additional point, arguing that screening only those at highest risk of disease is problematic where screening programs are associated with overdiagnosis, defined herein as detection of a cancer that would not otherwise have led to symptoms before a patient died of another cause. Specifically, a risk classifier that does not discriminate between disease incidence and disease mortality will not reduce overdiagnosis disproportionately. As a way forward, we introduce a simple formula for net benefit that incorporates both harms of screening—such as anxiety, or pain and side-effects of biopsy for false positives—and the harms of overdiagnosis, such as side-effects from treatment. We show that, if a marker predicts incidence rather than mortality, it will only be useful for determining a high-risk subgroup for screening under a limited number of scenarios, where the inherent harms of screening are high and the effects of screening on mortality are moderate. We recommend that research on use of PRS to inform screening should focus on SNPs associated with cancer mortality, rather than incidence.

We note that our emphasis on overdiagnosis is somewhat different from papers in the literature focusing on the screening “footprint”: screening programs are often evaluated in terms of the number of patients who need to be screened to prevent one death; here we also want to consider the number who are overdiagnosed. This work is therefore applicable to markers for cancers, such as breast and prostate, where overdiagnosis is associated with harm. Our findings are of less relevance for cancer screening programs where overdiagnosis is not an important problem, melanoma^6^ or colorectal cancer being obvious examples. Also note that we are specifically investigating the suggestion that PRSs be used to determine who and who not to screen^3,7,8^. This is quite separate from the proposal that PRSs determine the intensity of screening (e.g., annual vs. biennial) or age range (e.g., earlier starting for those with high PRS)^9^, approaches that are unlikely to make an important difference to overdiagnosis.

### Risk stratification for reducing the burden of screening

We will start by leaving aside the issue of overdiagnosis and consider what is arguably the more traditional approach, focusing only how a predictive marker could reduce the burden of screening. Although our interest is PRSs, we will use the term “marker” generically to refer to PRSs, as well as blood and imaging markers, clinical factors (such as age, race, age at menarche) or an algorithm that combines several predictors into a single score. Our reference strategy is a hypothetical population-based screening program that has been shown to increase diagnoses by 50 per 1000 and decrease mortality by 10 per 1000 (i.e., 40 overdiagnoses per 1000). We assume that a marker is developed to determine which members of the population should be screened and which exempted. If the marker is normally distributed on the logit scale and there is a 1 standard deviation difference between cancer cases and controls in the target population, the area-under-the-curve (AUC) would be around 0.75, with ~33%, 67%, and 80% of cancers found in the top 10%, 33%, and 50% of marker scores, respectively. Let us also assume that the marker does not predict mortality any better than incidence, that is, amongst those diagnosed with cancer, there is no difference in marker scores between those who do and do not subsequently succumb to cancer.

Although this is a hypothetical example, the parameters are close to estimates reported for PSA screening and prostate cancer PRSs, albeit a little favorable for the latter. A good estimate for PSA screening is that it leads to five additional cases per every prostate cancer death prevented^10^; a paper on a PRS reported that the proportion of cancers in the top 10%, 33%, and 50% of PRS risk approached 33%, 60%, and 80% respectively^11^; a different PRS was found to have almost identical hazard ratios irrespective of whether the endpoint was prostate cancer, aggressive prostate cancer, or prostate cancer death^12^.

We can now compare the strategy of screening all eligible individuals in the population with that of screening only those at high-risk. We must first assume that the probability an individual will develop cancer is independent of the probability early detection will prevent cancer-specific death. This seems to be a reasonable assumption and there is currently no evidence that, say, individuals with higher PRS scores are any more or less likely to have cancers incurable at screen detection. If we screen all eligible individuals, we need to screen 100 people to prevent 1 death; if we screen only those in the top 50%, 33%, or 10% of risk, the number of individuals screened to prevent one death is 63, 50, and 30, respectively (Table 1). While a threefold increase in risk for an individual undergoing screening seems like a vindication of risk-stratified screening, ratios are generally not helpful for decisions regarding delivery of population screening health.

**Table 1:** Net benefit of risk-stratified screening for the hypothetical reference case.

Strategy	Screen all	Screen 50%	Screen 33%	Screen 10%
Number screened per 1000	1000	500	333	100
Lives saved per 1000	10	8	6.7	3.3
Number screened to save one life	100	62.5	49.7	30.3
Maximum number of individuals we would screen to prevent one death				
1000	**9.00**	7.50	6.37	3.20
500	**8.00**	7.00	6.03	3.10
333	**7.00**	6.50	5.70	3.00
200	5.00	**5.50**	5.04	2.80
100	0.00	3.00	**3.37**	2.30
75	−3.33	1.33	**2.26**	1.97
50	−10.00	−2.00	0.04	**1.30**

Bold values denote the strategy with the highest net benefit.

A more traditional decision-analytic approach is to estimate net benefit, calculated as benefits minus harms, where harms are defined as all negative consequences of screening (such as anxiety, financial costs, biopsy for false-positives) and are weighted in terms of benefit^13^. For example, if we assume that an early death from cancer is 500 times more harmful than going through a screening program, the net benefit would be: lives saved minus individuals screened ÷ 500. Applying this formula to the numbers above we get a net benefit of 8, 7, 5.33, and 3.13 for screening all, 50%, 33%, or 10% of the population.

This result, that improving the ratio of patients screened per lives saved leads to worse outcome, appears to be counter-intuitive, but can be easily explained. For instance, we would prefer to give mammograms to 100,000 women and prevent 700 deaths than select only 100 women at highest risk and prevent 2 deaths, even though the latter strategy involves far fewer women screened per death avoided.

One obvious criticism of net benefit calculation is that there is room for reasonable disagreement over the relative harms of screening compared to a cancer-specific death. One researcher might stress the anxiety associated with false positives and the very real risks of biopsy; another might argue that such harms can be reduced by appropriate counseling and better biopsy technique. Hence, we can vary the “exchange rate” of the number of individuals we would be prepared to screen in order to prevent one cancer death. Table 1 gives the net benefit of screening strategies for various “exchange rates” and shows risk-stratified screening is only of greater net benefit than screening the full eligible population if screening is considered relatively harmful. For instance, if screening is thought to be only a 200th as bad as early death from cancer, the highest net benefit is obtained by screening only the 50% of the population at highest risk rather than screening the entire eligible population.

### Incorporating the harms of overdiagnosis

The full formula for the net benefit of a screening strategy (Eq. (1)), incorporating the harms of both screening and overdiagnosis, is given as:

1 $$Net\;benefit = Cancer\;deaths\;avoided-Overdiagnoses \div w_1-Individuals\;Screened \div w_2$$

where *w*_1_ and *w*_2_ are weighting factors. *w*_1_ is the relative harm of an overdiagnosis compared to a cancer death. It can be calculated by asking the question “What is the maximum number of individuals you would be prepared to diagnose with cancer in order to prevent one cancer-specific death?”. This is termed the “number willing to diagnose” or NWD. *w*_1_ is the NWD –1. *w*_2_ is the relative harm of screening—including all harms other than overdiagnosis—compared to a cancer death. It can similarly be calculated by asking “What is the maximum number of individuals you would be prepared to screen in order to prevent one cancer-specific death?”, the number-willing-to-screen, or NWS. *w*_2_ is NWS – 1, however, because NWS is normally high, the subtraction can generally be ignored. This gives Eq. (2):

2 $$Net\;benefit = Cancer\;deaths\;avoided-Overdiagnoses \div \left( {NWD-1} \right)-Individuals\;Screened \div NWS$$

Table 2 gives an overview of the harms associated with screening and those associated with overdiagnosis for some common cancer screening modalities, along with some illustrative NWD and NWS. For instance, the NWS is lower for lung computed tomography (CT) screening than for PSA because, while both types of screening can lead to painful biopsies in the event of a false positive, the actual procedure of lung CT, unlike a PSA blood test, is uncomfortable and involves risk. As a second example, the NWD is higher for the Pap smear than for mammography because treatment following a positive Pap test is far less harmful than surgery and chemotherapy for breast cancer. As pointed out above, NWD and NWS are a judgment call and can vary between researchers.

**Table 2:** Benefits and harms associated with screening in four common cancers.

Screening modality	Reduction in death per 1000 screened	Overdiagnosis per 1000 screened	Principal inherent harms of screening	Principal harms of overdiagnosis	Relative value of avoiding a death vs. undergoing screening (NWS)	Relative value of avoiding a death vs. overdiagnosis (NWD −1)
PSA^10^	10	40	Pain and risk of biopsy	Urinary and erectile dysfunction, side-effects of hormonal therapy	500	10
Pap smear^18^	8	200	Uncomfortable procedure	Side-effects of local treatment	1500	30
Mammography^17^	7	3	Uncomfortable procedure, need for follow-up imaging, pain of biopsy	Operative morbidity including poor cosmetic outcome, side-effects of chemotherapy and hormonal therapy	500	20
Lung CT^19,20^	3	20	Radiation exposure, uncomfortable procedure, pain and risk of biopsy	Operative morbidity, including chronic postoperative pain	350	15

Table 3 shows net benefit for various combinations of screening strategies, harm of screening and harm of overdiagnosis, using the reference strategy of a population-based screening program comparable to that for prostate cancer. If a marker does not distinguish between incidence and mortality, there are only a few scenarios in which screening a high-risk subgroup is of greater net benefit than screening the entire eligible population. If screening is relatively harmful (i.e., the NWS and NWD are low), then the “screen all” strategy has negative net benefit, and in some of these cases, screening a small subset of the population, such as the top 10% at risk, is sometimes a preferable strategy. There are also some cases where net benefit is positive for the “screen all” strategy but there is higher net benefit from screening the top 50% of risk. Table 3 also gives net benefit when the discrimination of the marker is 0.65 rather than 0.75, which is closer to what has been reported for PRSs in many studies, for example, breast cancer^14,15^. Risk stratified screening is rarely favored in this scenario. An Excel spreadsheet in the Supplementary Material allows users to enter their own parameters to see effects on net benefit.

**Table 3:** Net benefit of risk-stratified screening for the reference case, accounting for the harms of the screening test across different scenarios.

NWS	NWD-1		Marker does not discriminate between incidence and mortality				Marker discriminates between incidence and mortality			
		Screen All	Screen 50%	Screen 33%	Screen 10%	Highest net benefit for less predictive marker	Screen 50%	Screen 33%		Screen 10%
750	30	**7.33**	6.27	5.37	2.73	5.40	**7.77**	7.73	5.92	
750	20	**6.67**	5.73	4.92	2.51	4.93	7.23	**7.26**	5.65	
750	10	**4.67**	4.13	3.58	1.85	3.53	5.63	**5.86**	4.83	
500	30	**6.67**	5.93	5.15	2.66	5.07	7.43	**7.51**	5.85	
500	20	**6.00**	5.40	4.70	2.44	4.60	6.90	**7.04**	5.58	
500	10	**4.00**	3.80	3.36	1.78	3.20	5.30	**5.64**	4.76	
250	30	4.67	**4.93**	4.49	2.46	4.07	6.43	**6.85**	5.65	
250	20	4.00	**4.40**	4.04	2.24	3.60	5.90	**6.38**	5.38	
250	10	2.00	**2.80**	2.70	1.58	2.20	4.30	**4.98**	4.56	
100	20	−2.00	1.40	**2.06**	1.64	0.70	2.90	4.40	**4.78**	
50	20	−12.00	−3.60	−1.24	**0.64**	−0.40	−2.10	1.10	**3.78**	
25	20	−32.00	−13.60	−7.84	−1.36	−2.40	−12.10	−5.50	**1.78**	
1500	30	**8.00**	6.60	5.59	2.79	5.73	**8.10**	7.95	5.99	
2500	50	**8.80**	7.16	6.03	3.00	6.24	8.66	8.41	6.23	

Bold values denote the strategy with the highest net benefit.

The results for a marker that predicts mortality better than incidence are also shown in Table 3 (columns to the right). An example of such a marker is PSA, which, in a long-term study of 1167 men aged 60 not subject to screening^16^, had a much higher AUC for prostate cancer death (0.90) than for prostate cancer incidence (0.76), with the proportion of cases/deaths in the top 50%, 33%, and 10% of PSA levels being 80%/95%, 70% / 91%, and 41% / 66%, respectively. Use of a marker with these properties to determine eligibility for screening always has superior net benefit to a marker than predicts incidence and mortality equally. It is superior also to a strategy of screening the entire population, except in the unusual case where screening is extremely benign, where we would be willing to screen over 2000 patients to prevent one death.

Table 4 shows the effects of risk-stratified screening for PSA^10^, mammography^17^ and pap smear^18^, using empirical estimates from the literature for overdiagnosis and mortality reduction, and the authors’ opinions on the harms of screening and overdiagnosis relative to cancer mortality. A marker that does not discriminate between incident cancer and fatal cancer is only of greater net benefit than the “screen all” strategy for Pap smear and one of the mammography scenarios—where the inherent harms of screening are considered to by high—moreover, the absolute differences are small, and are lost for marker with lower discrimination. A marker that has a higher discrimination for lethal compared to incident cancer has highest net benefit for PSA, Pap smear and lung CT, but only one of the mammography scenarios. However, again, there is limited benefit to risk stratifying in any scenario if the discrimination of the marker is lower (AUC of 0.65 for lethal disease). If AUC of the marker for mortality is higher (0.825 or above), risk-stratification is of benefit even for the mammography scenarios (see Supplementary Material).

**Table 4:** Net benefit of risk-stratified screening for common cancer screening approaches for a marker that does not distinguish between incidence and mortality.

Screening approach	NWS	NWD-1		Marker AUC = 0.75				Marker AUC = 0.65			
				Marker that does not discriminate between mortality and incidence			Highest net benefit for marker that discriminates between mortality and incidence	Marker that does not discriminate between mortality and incidence			Highest net benefit for marker that discriminates between mortality and incidence
			Screen all	Screen 50%	Screen 33%	Screen 10%		Screen 50%	Screen 33%	Screen 10%	
PSA	500	10	4.00	3.80	3.36	1.78	**4.20**	3.20	2.34	1.00	3.60
Pap Smear	1500	30	0.67	**0.73**	0.67	0.37	**1.40**	0.60	0.45	0.20	**1.27**
Mammography	500	20	4.85	4.48	3.93	2.06	4.50	3.80	2.77	1.17	3.81
Mammography (alternative I)	350	15	3.94	**4.01**	3.61	1.96	**4.03**	3.33	2.46	1.07	3.35
Mammography (alternative II)	500	10	4.70	4.36	3.83	2.01	4.39	3.69	2.69	1.14	3.72
Lung CT	350	15	−1.19	−0.10	**0.17**	**0.26**	**0.44**	-	-	-	-

Bold values denote the strategy with the highest net benefit.

We then expanded our analysis by plotting net benefit against the full range for the proportion screened, using the scenario of PSA screening and a marker that does not distinguish between incidence and mortality (see Supplementary Fig. 1). We found net benefit could be very slightly increased if we exclude from screening a small proportion of patients at particularly low risk. However, there are several reasons to believe that this is a somewhat misleading finding. Firstly, it is only seen for a marker with an AUC of around 0.75, higher than seen for current PRSs. Secondly, the very slight increase in net benefit, around 0.17 per thousand, is likely offset by the loss in net benefit associated with cost and anxiety of giving the PRS. Third, and perhaps most critically, the harms of screening incorporated in calculation of net benefit include those associated with false positives, such as pain and risk of biopsy. However, it is likely that the false positive rate will show at least a slight positive correlation with risk score. For example, a SNP that increases inflammation may increase the risk of both prostate cancer and benign conditions that raise PSA. Hence, excluding from screening patients in the bottom 25% of risk is unlikely to avoid sufficient false positives to favorably influence outcomes of a screening program.

Results for lung CT screening are given in Table 4 as an example of a cancer screening modality currently offered only to a subgroup of the population^19,20^. In the EPIC study of 169,035 ever smokers aged 40–65, the discrimination of smoking history—how eligibility for lung CT is currently determined—is close to 0.75^21^. The key point is that screening all eligible patients (or even the top 50% of risk) has negative net benefit, that is, it does more harm than good. Screening the top 10% at risk is the only strategy associated with non-trivial positive net benefit. This accords approximately with our current practice of offering CT scans only to patients with a significant smoking history, there being no serious suggestions to make lung cancer screening a population-based intervention.

Above and beyond our net benefit calculations, our primary conclusion can be explained heuristically. In the absence of concerns about overdiagnosis, risk-based screening is not of value if the harms and costs of screening are low, because then population-based screening allows us to detect all or most of the cancers. Risk-based screening becomes more efficacious as the relative harms of screening increase, and also as the accuracy of the marker (such as a PRS) increases, since a relatively accurate marker allows us to minimize the harms of screening while detecting a relatively large proportion of the cases. Similar considerations apply for cancers where overdiagnosis is a concern: risk-based screening can only offset harms to the extent that the marker is accurate in the sense that it distinguishes patients who are likely to die of their disease from those who are overdiagnosed. In the absence of such discrimination, restricting screening to a subgroup at higher risk reduces benefits more than it reduces harms. Consequently, in the setting of diseases where overdiagnosis harms are of particular concern, a strategy that restricts screening to a high-risk subgroup will only be of benefit if a PRS has high accuracy for identifying lethal cancer and superior discrimination between lethal and non-lethal disease.

### Published research on the effect of PRS-stratified screening

Several studies have used simulation studies and purport to show that stratifying screening using a PRS is likely to improve cancer outcomes ^7–9,22^. Recently, Callender et al. claimed that “Screening men at a higher risk of prostate cancer [as assessed by a PRS] lowers the ratio of overdiagnosed cases to prostate cancer deaths averted … leading to an improvement in the benefit–harm profile as the risk threshold rose”^7^. A cost-effectiveness study on breast cancer similarly claimed that restricting mammography to women at higher risk from a PRS could have a large impact on overdiagnosis, with up to a 70% decrease in avoidable diagnoses, but minimal impact on lives saved (~10% reduction)^22^. The authors of these papers have yet to provide a mechanism by which a PRS reduces overdiagnosis while simultaneously preserving number of lives saved, despite correspondence suggesting errors in their mathematical approach^23^.

BARCODE1 is an empirical pilot study of implementing a PRS to risk stratify screening and has recently published initial findings^3^. Of 1434 men sent a written invitation, 297 provided usable samples for genotyping; 25 were found to be at high risk, 18 of whom underwent a prostate biopsy, with 7 low-grade (overdiagnoses) but no high-grade cancers found. The PRS used in BARCODE1 study includes 130 risk SNPs that associate with prostate cancer incidence; presently no risk loci specific for aggressive prostate cancer are included. This may explain the reason for what is clearly a very disappointing result for genetic risk stratification.

## Conclusions

Contemporary PRSs have primarily been developed for the endpoint of cancer incidence. If these PRSs do not discriminate lethal from non-lethal disease, using them to determine who to subject to a screening strategy associated with overdiagnosis is unlikely to be of benefit compared to our current strategy of screening the entire eligible population. It may be that contemporary PRSs could be of benefit for determining age range or screening interval, as exemplified by the WISDOM study^24^, which evaluates biennial rather than mammography and starting screening at 50 rather than 40 for women at low risk. Nontheless, we advocate that research on PRS focus on cancer mortality, which is currently relatively unchartered. In particular, developing PRSs that have higher discrimination for cancer mortality rather than for cancer incidence will be a significant advancement for improving screening outcomes.

## Supplementary information

Supplementary Figure 1

Supplementary Data

## Data Availability

No empirical data were analyzed for this study.

## Code availability

The study findings were generated using simple formulae in Microsoft Excel v16.5, rather than code. The spreadsheet is included as an attachment. There are no restrictions on access.
