# Supplementary figures and images for: Polygenic risk scores to stratify cancer screening should predict mortality not incidence
Source: NPJ Precis Oncol. 2022 May 30;6:32. doi: 10.1038/s41698-022-00280-w (PMC9151796; doi:10.1038/s41698-022-00280-w)

Net benefit per thousand

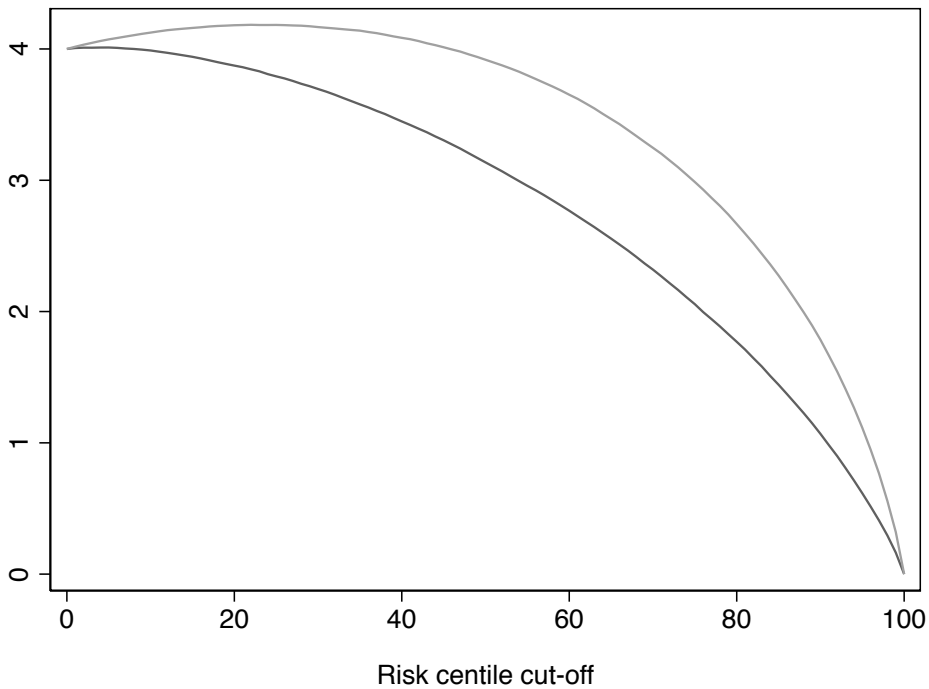

Supplement: Supplementary file 1 — Supplementary Figure 1 [file 41698_2022_280_MOESM1_ESM.pdf]
